# Do ‘environmental bads’ such as alcohol, fast food, tobacco, and gambling outlets cluster and co-locate in more deprived areas in Glasgow City, Scotland?

**DOI:** 10.1016/j.healthplace.2018.04.008

**Published:** 2018-05

**Authors:** Laura Macdonald, Jonathan R. Olsen, Niamh K. Shortt, Anne Ellaway

**Affiliations:** a MRC/CSO Social and Public Health Sciences, University of Glasgow, Top floor, 200 Renfield Street, Glasgow G2 3QB, United Kingdom; b Centre for Research on Environment, Society and Health, School of Geosciences, University of Edinburgh, Drummond Street, Edinburgh EH8 9XP, United Kingdom

**Keywords:** Cluster analysis, Area deprivation, Alcohol, Fast food, Tobacco, Gambling

## Abstract

This study utilised an innovative application of spatial cluster analysis to examine the socio-spatial patterning of outlets selling potentially health-damaging goods/services, such as alcohol, fast food, tobacco and gambling, within Glasgow City, Scotland. For all categories of outlets combined, numbers of clusters increased linearly from the *least* to the *most* income deprived areas (i.e. one cluster within the least deprived quintile to ten within the most deprived quintile). Co-location of individual types of outlets (alcohol, fast food, tobacco and gambling) within similar geographical areas was also evident. This type of research could influence interventions to tackle the co-occurrence of unhealthy behaviours and contribute to policies tackling higher numbers of *‘environmental bads’* within deprived areas.

## Highlights

• For all outlets combined, cluster numbers increased with increasing deprivation.
• Poorer areas showed more alcohol, fast food, tobacco, and gambling outlet clusters.
• Our evidence suggested co-location of various types of outlets in similar areas.
• Cluster analysis was used as an innovative tool to explore spatial retail access.

## 1 Introduction

Health-related behaviours, such as smoking, heavy drinking and poor diet, can lead to higher risk of chronic disease, multi-morbidity, and shortened life span (Cawley and Ruhm, 2011, Fortin et al., 2014). The drivers of such behaviours are multifactorial and recent work has acknowledged that health behaviours are influenced, not just by personal attributes, but also by features of the broader physical, social, economic and cultural environments (Shortt et al., 2014). Most recently there has been a focus on the retail environment specifically, and the ways in which it may contribute to the health ‘chances’ afforded to the population (Thomas and Frohlich, 2012). With a focus on inequalities, research has begun to explore the relationship between such environmental risk factors and area level deprivation.

Recent research has explored the distribution of each of these outlet types in isolation: tobacco (Shortt et al., 2015, Chaiton et al., 2013), alcohol (Hay et al., 2009, Ellaway et al., 2010), fast food (Fraser et al., 2010, Macdonald et al., 2007), and gambling outlets (Robitaille and Herjean, 2008, Wardle et al., 2014). It has been reported that an increased availability of each is related to an increase in related unhealthy behaviours; for smoking (Reitzel et al., 2011, Novak et al., 2006, Shortt et al., 2016), increased alcohol consumption (and related violence) (Connor et al., 2010, Livingston, 2008, Young et al., 2013), increased consumption of fast food and increased obesity rates (Bodicoat et al., 2015, Moore et al., 2009) and increased likelihood of problem gambling (Pearce et al., 2008, Young et al., 2012). Whilst the health consequences of smoking, excess alcohol consumption and an unhealthy diet are well established, for gambling the association with health is under-researched (Lancet Editorial, 2017). Problem gambling has however been linked to several health outcomes, such as increased alcohol consumption, obesity, smoking, mental health problems, and suicide (Barnes et al., 2015a, Black et al., 2013), as well as intimate partner violence (Afifi et al., 2010). Although the majority of work in this area has focussed on features of the environment in isolation, unhealthy behaviours do interact (such as alcohol misuse and smoking) (Buck and Frosini, 2012, Meader et al., 2016, Room, 2004) and individuals do not experience one type of retail outlet in isolation from the others.

Strong socioeconomic gradients in retailer presence exist within the UK and further afield, and it has been suggested that the overprovision of a range of health damaging outlets in deprived areas is a form of *‘environmental injustice’* (Mennis et al., 2016, Romley et al., 2007). Within Scotland, compared to more affluent areas, deprived areas showed greater densities of alcohol, tobacco and gambling outlets (Shortt et al., 2015, Wardle et al., 2014); in Canada tobacco outlet and gambling outlet densities were higher within deprived areas (Chaiton et al., 2013, Wilson et al., 2006); and deprived areas in New Zealand and Australia showed higher densities of alcohol outlets and fast food outlets respectively (Hay et al., 2009, Thornton et al., 2016). Such gradients may contribute to the wider social gradient in health related outcomes. This association is not always clear-cut and findings vary by geographical region. For example, earlier Glasgow-based studies found no clear socio-spatial patterning in the distribution of fast food or alcohol outlets by deprivation (Ellaway et al., 2010, Macintyre et al., 2008).

Limited work looks at the availability of a variety of categories. One study explored the availability of three types of retailer, tobacco, alcohol and takeaway outlets in four districts in Cologne, Germany (Schneider and Gruber, 2012), while other research calculated the density of alcohol and tobacco outlets in small neighbourhoods across Scotland (Shortt et al., 2015); both studies compared access between areas with varying levels of income deprivation. We go beyond previous work by exploring four categories of outlets offering potentially harmful products/services (both individually and in combined analysis), recognising that people are exposed to multiple characteristics day-to-day. We focus specifically on locating ‘clusters’ of outlets (i.e. occur closely together) and explore whether these ‘co-locate’ (i.e. different categories of outlet found in similar areas) within poorer neighbourhoods. Previous research used a more traditional approach of comparing densities across geographical areas (Chaiton et al., 2013, Ellaway et al., 2010, Thornton et al., 2016, Wiggins et al., 2010, Shortt et al., 2016, Hay et al., 2009, Wardle et al., 2014, Wilson et al., 2006). Within these studies densities of outlets were generally calculated for pre-defined geographical and administrative boundaries e.g. density of alcohol outlets across Scottish ‘Data Zones’ (Ellaway et al., 2010), fast food outlet density at Australian ‘Local Government Area’ (Thornton et al., 2016), density of tobacco outlets at Canadian ‘Public Health Unit’ level (Chaiton et al., 2013), or gambling outlet density within US ‘Census Tracts’ (Wiggins et al., 2010). The benefit of this approach was the potential to link these boundaries to additional key data such as poverty rates (Ellaway et al., 2010, Thornton et al., 2016), indicators of urbanicity/rurality (Thornton et al., 2016), population ethnicity (Wiggins et al., 2010), or number of smokers (Chaiton et al., 2013). The cluster analysis approach applied within our paper has previously been used to detect geographic disparities in the incidence of disease cases, e.g. cancer (Goungounga et al., 2016), tuberculosis (Roth et al., 2016) and HIV (Zhang et al., 2017, Goungounga et al., 2016, Roth et al., 2016) and to explore socio-economic distribution of road traffic accident cluster locations following the construction of a new motorway (Olsen et al., 2017). Spatial cluster analysis has rarely been used to identify concentrations of retail outlets (Han and Gorman, 2014) but lends itself well to this type of study for a number of reasons. Primarily, it is a form of dynamic mapping which is not restricted by pre-defined boundaries but locates natural concentrations of outlets. In doing so it provides objective, robust detection of potential retail clusters. Furthermore, it enables the detection of small area levels of groups of outlets containing higher than expected cases rather than applying a smoothed density surface to a pre-defined geographical area such as a census tract.

We examine the distribution of alcohol, tobacco, fast food and gambling outlets within the geographical context of Glasgow because the city contains areas of stark contrast, consisting of the most and least deprived areas in Scotland with nearly half (48%) of neighbourhoods falling within the 20% most income deprived areas in Scotland (The Scottish Government, 2016a). Glasgow displays an adverse health profile in comparison to the rest of Scotland (Gray, 2008), and compared to those residing within similar cities with similarly high levels of socio-economic deprivation (Walsh et al., 2010). The current study furthers our previous work on the socio-spatial patterning of retail outlets and other amenities within Glasgow City and across Scotland by using a novel application of cluster detection to advance the field. Our earlier work looked at the density of food outlets and alcohol outlets across pre-defined geographical boundaries (i.e. small area level geography known as data zones), linking this geography to deprivation scores and comparing more or less deprived areas within Scotland (for fast food chains such as McDonald's (Cummins et al., 2005) Burger King, KFC and Pizza Hut (Macdonald et al., 2007)), and Glasgow (for various out of home food outlets (Macintyre et al., 2005), food retailers (Macdonald et al., 2009), amenities (e.g. schools, leisure centres, hospitals) (Macintyre et al., 2008), and alcohol outlets ((Ellaway et al., 2010), (Young et al., 2013)).

The main objectives of this research are to explore whether particular areas are subject to excess access to potentially health damaging retailers and whether these types of retailers co-locate within these areas. We do this by examining the socio-spatial patterning of a range of retail outlets which sell potentially health damaging products (alcohol, tobacco, fast food) or services (gambling) in combination and separately; utilising an innovative application of cluster analysis to detect if geographic clusters of these outlets exist (i.e. outlets locate closely together) and co-locate (i.e. different categories of outlet found in similar areas) within poorer neighbourhoods.

## 2 Methods

### 2.1 Outlet data

Address data for all outlets were obtained from Glasgow City Council (i.e. the local government body for the City of Glasgow), for 2012 (tobacco and fast food), and 2013 (alcohol and gambling). Although we did not validate address information for every outlet, due to the number of premises, the data held is deemed as comprehensive as information on the various premises is required to be held by Glasgow City Council for inspection, planning and licensing purposes (see Ellaway et al., 2010, 2012) (e.g. food premise/standards inspection, planning permission for gambling outlets, alcohol premise licensing, tobacco retailers register).

The types of outlets in the current analysis included: 1) alcohol outlets (including off-sales (off licence stores, convenience stores, and supermarkets) and on-sales (restaurants, cafes, public houses, hotels, nightclubs, entertainment venues, social and sports clubs)); 2) fast food outlets: fast food chains, premises selling fast food (e.g. Chinese food, Indian food, burgers, kebabs, fish and chips, pizza etc.); 3) tobacco outlets: convenience stores, newsagents, supermarkets, petrol stations, off licence stores; and 4) gambling outlets: betting shops, lottery vendor, bingo halls, casinos, gambling machines. The postal codes for the outlets were linked to precise geo-coordinates via the Office for National Statistics Postcode Directory (for August 2011) which contains British National Grid coordinates for address-weighted unit postcode centroids (Office For National Statistics, 2016). Data cleaning included checking for duplicates and correcting postcodes which did not map.

### 2.2 Analysis

#### 2.2.1 Detection of outlet clusters

**Fig. 1:**
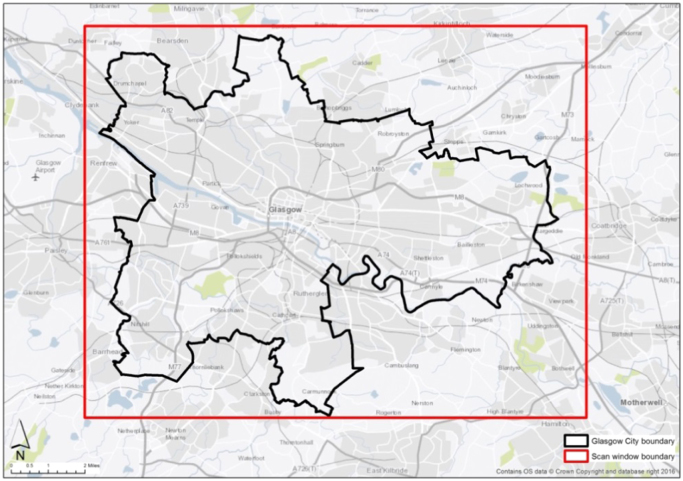
Glasgow city and spatial scan window boundary.

SaTScan™ is a well-established cluster analysis tool that allows for a variety of spatiotemporal cluster analyses based on various probability models. The procedure can identify geographically defined clustered areas of high risk, low risk, or both, for the occurrence of retail outlets, within a defined geographical boundary, enabling each individual cluster to be compared to the whole geographical area in question (Kulldorff, 2010). The software constructs a large number of different sized circular frames (from zero to an imposed upper limit, *specified below*) with varying location and radii across the study area and then makes a comparison of occurrence of outlets within each frame and the occurrence outside the frame. The close location of frames with apparently higher rates of outlets is used to identify the location and size of a cluster, and its statistical significance is then determined (i.e. this method identifies regions that are significantly different from neighbouring regions) (Duncan et al., 2016). The scan window used for the analysis surrounded that of Glasgow City, the boundary is supplied in Fig. 1, and we chose this rectangular boundary as we have previously conducted sensitivity analysis of this window size (Olsen et al., 2017).

For each circular frame the software tests the null hypothesis of constant risk of outlets throughout the area with the alternative hypothesis that there is an elevated risk within the circular radii outside of it. For cluster detection, we used a *continuous Poisson-based* model in SaTScan™ v.9.4.2 (Satscan, 2005) to detect spatial clusters of georeferenced outlets; the assessment of a cluster was made using a likelihood ratio test. The model uses a space-time permutation model that is useful when only case or count data are available, as is the case for our dataset (Sparks et al., 2012). We used a continuous Poisson-based model due to the ability to measure the location of retailers as random locations across the geographic boundary, other procedures within SaTScan consider locations to be non-random or ‘yes/no’ in terms of a point location containing an outcome or not; we only included point data if they were of interest (i.e. an alcohol retailer). It allowed us to test whether the locations of retailers in the study window were randomly distributed spatially or whether there were clusters of retailers. The benefit being the procedure tests the null hypothesis that the retailers follow a homogenous spatial Poisson process with constant intensity throughout the study area, the procedure uses a circular scanning window as this model has not been implemented to use an elliptic window (Satscan, 2005). We did not apply population weighting to analysis as this allowed us to use a purely spatial detection of retailers. We believe it is important these clusters are detected regardless of whether there are a greater number of people residing within that area, as this may equate to greater population level exposure.

Analysis was performed for all four categories of outlets combined, to examine co-location, and for alcohol, fast food, tobacco, and gambling outlets individually. The software provided an output of the centroid of each cluster, its size (radius) and its statistical significance. Significant (p < 0.05) clusters detected in SaTScan™ were mapped using ArcMap version 10.3 to display their centroids and sizes. We applied a cluster limit of 500 Cartesian units (i.e. spatial units based on the Cartesian coordinate system which specifies each point uniquely in a plane by a pair of numerical coordinates) for the statistical analysis; it is considered good practice that clusters are made as small as possible to ensure that low risk neighbourhoods are not incorrectly included in a larger high-risk area as it is possible to sustain statistical significance over a large geographical area which can encompass low risk areas (Penna et al., 2009). We chose the upper bound limit for cluster of 500 Cartesian units, which equalled a diameter limit of 1 km (kilometre) and 0.5 km from the centroid of the cluster, which is a commonly used measure of reasonable walking distance within physical activity research (Laverty et al., 2015).

### 2.3 Linking clusters to income deprivation

We obtained a look-up table linking data zones (i.e. small-area statistical geography containing between approximately 500 and 1000 residents (The Scottish Government, 2005)) to the Scottish Index of Multiple Deprivation 2012 (SIMD) Income sub-domain score; the income score is based on numbers of claimants for a range of welfare benefits e.g., Income Support, Jobseekers Allowance, Tax Credits etc. (The Scottish Government, 2012). We chose not to use the full SIMD as it includes drive time to amenities within the ‘Access’ sub-domain; associating access to the outlets included in this analysis with a measure that included the Access domain would be tautological (Dundas et al., 2007). Data zone Income scores were grouped into quintiles (Q1: most deprived, Q5: least deprived). Maps of geographic centroids of data zones and cluster boundaries were overlaid and where a data zone centroid fell within a cluster boundary it was linked to that cluster. Mean SIMD Income scores were calculated for data zones within each cluster, allowing each cluster to be allocated an income score/quintile.

## 3 Results

Within Glasgow there were 1718 alcohol outlets, 903 fast food outlets, 870 tobacco outlets and 262 gambling outlets. Table 1 contains the number of statistically significant retail outlet clusters; 28 clusters for all outlets combined, 20 for alcohol outlets, 16 for fast food outlets, 15 for tobacco outlets and 5 for gambling outlets. Clusters are distributed across a range of areas in terms of the most and least deprived but skewed towards the most deprived areas with few in the least deprived areas. For all outlets, alcohol, fast food, tobacco and gambling outlets the most deprived areas of Glasgow contain the largest number of clusters. See Supplementary Table 1 for the full output containing test statistics for each cluster by SIMD quintile, i.e. mean observed and expected counts, log likelihood ratio (provides evidence of the elevated risk of a retail outlet in that area) and p-values.

**Table 1:** Number of spatial clusters by retailer and SIMD Income quintile.

	**Number of clusters**				
**SIMD Income quintile**	**All outlets**	**Alcohol outlets**	**Fast food outlets**	**Tobacco outlets**	**Gambling outlets**
**1 (most deprived)**	10	6	6	7	2
**2**	8	6	4	2	1
**3**	6	4	4	4	1
**4**	3	3	1	1	1
**5 (least deprived)**	1	1	1	1	0

**Fig. 2:**
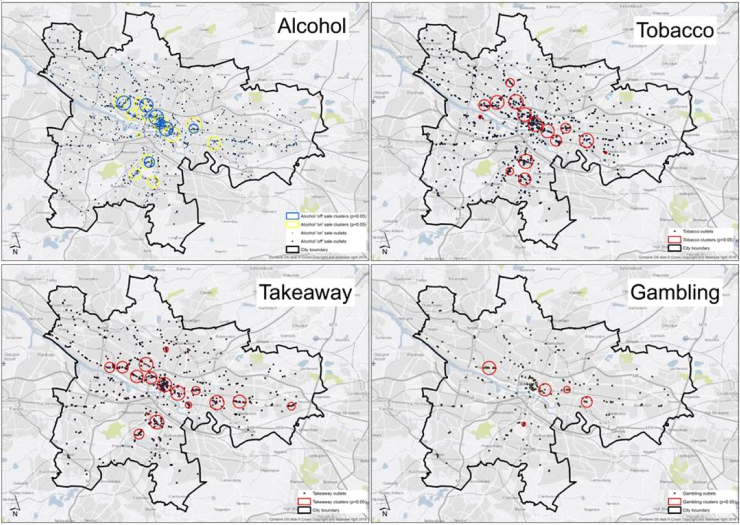
Spatial clusters of outlets by retailer, Glasgow City.

**Fig. 3:**
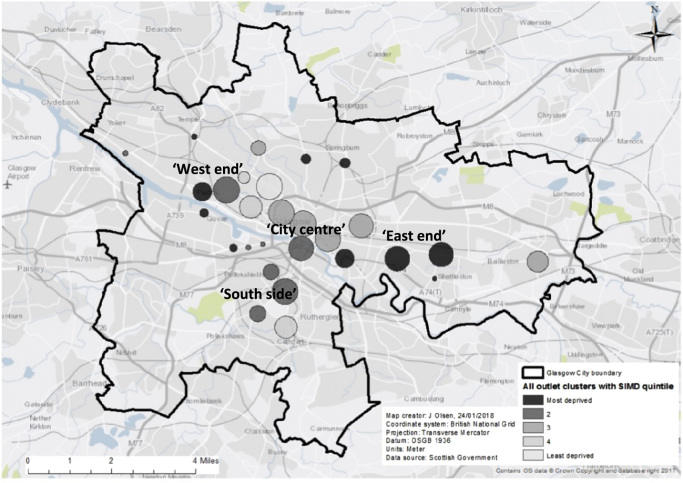
Clusters of outlets (all) by SIMD Income quintiles, Glasgow City.

Fig. 2 shows the location of outlets and clusters for each of the outlet categories. Fig. 3 shows the distribution of outlet clusters for all outlet types by socio-economic status (SIMD Income quintiles; clusters in most deprived areas in black, least deprived in light grey). The maps show that although there is variation in the location of the various categories there is evidence of co-location of clusters in similar geographical areas; generally the clusters are located in the central business district (i.e. the city centre), other retail, office and service hubs in the ‘west end’ and ‘south side’ (i.e. south of the River Clyde), and also areas in the ‘east end’. Fig. 3 shows clusters of varying radii; a small radius indicating a small concentration of outlets, compared to a larger frame or a large number of frames in close proximity. A number of the cluster radii cover areas displaying a range of deprivation scores; however some radii within the east end of the city include mostly deprived areas only.

## 4 Discussion

The aim of this study was to use a novel application of spatial cluster analysis to explore the socio-spatial patterning of retail clusters in Glasgow, Scotland. We believe that the form of dynamic mapping used here, where spatial analysis is not restricted by pre-defined boundaries but locates natural concentrations of outlets, advances existing methods for quantifying spatial access to retail outlets. Our findings indicate a greater number of outlet clusters located within more deprived areas; all outlets (combined), alcohol outlets, fast food outlets, and tobacco outlets were clustered within the most deprived areas, while only slightly more clusters of gambling outlets were located within the most deprived areas.

These findings highlight the need to go beyond focusing on clusters of individual categories of outlets, as in existing research, to explore not only clusters of a range of different categories of outlets but also co-location of such clusters within similar areas (as seen in Fig. 2, Fig. 3). This type of research has the potential to benefit the study of the mechanisms that drive health behaviours deemed ‘complementary’ (i.e. often occur together) such as alcohol and tobacco use (Room, 2004) and implications for interventions to tackle both. Indeed an intervention to reduce smoking within Scotland by prohibiting tobacco use in pubs and bars also led to reduction in alcohol consumption in moderate/heavy drinking smokers (Mckee et al., 2009). To reduce the impact of alcohol and tobacco availability in society it is important to understand the mechanisms that drive the use of both, in *isolation* and in *combination*. This could extend to other health behaviours that may co-occur such as drinking and problem gambling (Welte et al., 2004). Living in an environment with more availability of either product, or a combination of both, may increase their use and undermine attempts to reduce either or both behaviours. Examining the availability of related products may provide us with an improved understanding of the role of the retail environment in multiple health behaviours.

### 4.1 Do outlets selling unhealthy products cluster in deprived areas?

For all categories of outlets, the numbers of clusters increased linearly from least to most deprived quintiles with one cluster located in Q5 (least deprived) compared to ten within Q1 (most deprived). There appear to be areas which show concentrations of a number of different types of outlets selling unhealthy services, suggesting co-location. Loomis et al. remarked upon the tendency for poorer areas to not only have greater access to tobacco outlets, but also to other potentially health damaging services such as alcohol and fast food outlets (Loomis et al., 2013), however their research did not explore whether various categories of outlet co-located within deprived neighbourhoods. Previous research in Scotland (Shortt et al., 2015) found that outlets selling tobacco and off-sales were co-located in deprived areas, with these areas exhibiting the highest densities of both. Similarly, a study based in Cologne, Germany, found that clusters of alcohol, fast food, and tobacco were more likely located within low income areas thus creating potentially “addictive environments” through a ‘culmination’ of health damaging exposures for residents (Schneider and Gruber, 2012).

### 4.2 Do individual types of outlets co-locate in deprived areas?

There was evidence of co-location of the individual types within similar geographical areas (see Fig. 2, Fig. 3); many of the different categories of clusters were located within the same areas i.e. around busy main roads, and major shopping thoroughfares, with some clusters based in areas of particularly high deprivation within the east end of the city. This co-location of outlets may reflect shopper convenience (i.e. everything they need in one area), or another theory is that retailers purposefully choose areas close to populations with greater demand for specific goods such as alcohol, fast food, tobacco etc. but also choose the areas where retail rent is lower i.e. disadvantaged areas. Evidence for this was found for the siting of alcohol outlets within Melbourne, Australia (Morrison et al., 2015). However from our study we cannot ascertain *why* outlets were co-located within deprived areas.

Alcohol outlets were found to cluster in the most deprived areas. The results from our Glasgow-based research suggest a similar pattern compared to studies undertaken within Australia (Livingston, 2012), New Zealand (Hay et al., 2009), and the US (Gorman and Speer, 1997) which show a higher concentration of alcohol outlets in deprived areas. Previous research within Glasgow indicated that alcohol outlets were not necessarily located within deprived areas, (Ellaway et al., 2010), however this research used a different measure of geographical analysis (i.e. density analysis) and did not use a more complex measure to locate clusters of outlets as seen in this current work. A previous study which did make use of the spatial scan technique to explore geographic clustering of alcohol outlets in Lubbock, Texas found on-sales to be clustered within a particularly deprived area while off-sales were dispersed across Lubbock and located along major highways and roads which the authors believed reflected the large numbers of drivers within Texas (Han and Gorman, 2014). Han and Gorman (2014) emphasised the importance of considering local context when studying spatial access to alcohol; indeed for Glasgow the presence of clusters of alcohol outlets in the east end could be related to its history of industry, ‘working class’ life, and to this day high deprivation.

The most deprived areas of Glasgow contained a greater number of fast food clusters and tobacco outlet clusters, compared to the least deprived areas. This corresponds to previous fast food outlet research across Scotland and Glasgow City (Shortt et al., 2015), Canada (Chaiton et al., 2013), the US (Loomis et al., 2013, Yu et al., 2010), Australia (Wood et al., 2013) and New Zealand (Marsh et al., 2013), and to tobacco outlet work within England and Scotland (Macdonald et al., 2007), the US (Reidpath et al., 2002), Australia (Thornton et al., 2016) and New Zealand (Pearce et al., 2007). Gambling outlets were the least prolific of the retail categories studied and showed the lowest number of clusters (n = 5), although the most deprived quintile displayed the greatest number (n = 2) and the least deprived areas had none. Similarly, existing studies of the distribution of gambling machines observed positive associations with area income deprivation across the UK (Wardle et al., 2014), and Canada (Wilson et al., 2006). It could be argued that the physical presence of a gambling outlet may be less important to health behaviours due to the proliferation of on-line gambling, nonetheless existing research found that an increased availability of gambling outlets was related to crime, anti-social behaviour and other environmental incivilities (Bradford, 2011) and an increased likelihood of problem gambling (Pearce et al., 2008, Young et al., 2012). One particular US study found that those living within three minutes of a lottery outlet where twice as likely to suffer from problem gambling, and three times as likely to gamble frequently, than those living greater than ten minutes away (Welte et al., 2006).

### 4.3 Policy/licensing implications

Our findings provide some support for constraints on alcohol, fast food, tobacco and gambling outlets location in areas subject to overprovision such as low income neighbourhoods. In terms of licensing of alcohol outlets there are numerous controls put in place by local authorities which aim to deal with crime, disorder and public nuisance, public health, public safety, child safety etc. (City Of Glasgow Licensing Board, 2016). However, various clusters do exist within Glasgow City (n = 18), and with the established association between high concentrations of alcohol outlets and increased risk of anti-social behaviour, such as alcohol-related assaultive violence (Grubesic and Pridemore, 2011, Livingston, 2008), there is a need for on-going assessment of policies/restrictions on alcohol outlet access.

Restrictions on fast food access are near invisible in UK policy (Foresight, 2007) with no guidance at a national level, and in a similar vein, within Scotland there is no legislation on tobacco retail density and no official licensing scheme. Retailers must sign the Tobacco Retailers Register (The Scottish Government, 2017) but need not pay a fee or meet specific requirements. Although the Scottish Government created a number of new laws to tighten the sale of tobacco (The Scottish Government, 2016c) there are no specific restrictions on clustering or overprovision of tobacco outlets within a neighbourhood. There is potential for local authorities to develop their own regulations to enhance health promoting influences within local areas, as seen in London borough Tower Hamlets with additional restrictions put on fast food availability (Caraher et al., 2013). Policy measures to reduce concentrations of fast food and tobacco outlets in areas which are subject to overprovision could include restrictions on new outlets being opened until a specified target is met, imposing minimum distance requirements between outlets, maintaining low proportions of outlets (e.g. less than five percent of all retail units as fast food/tobacco retailer), no outlets within specified boundaries of schools, and restrictions on opening hours (Ackerman et al., 2017, Caraher et al., 2013, Cohen and Anglin, 2009).

The overprovision of gambling outlets is a key policy concern; for example, as of February 2017, in Scotland planning permission must be achieved by those seeking to open (or change an existing unit) to a new gambling outlet (The Scottish Government, 2016b). Although individual local authorities, such as Glasgow City Council, recognise that issues such as loss of retail function and vacancy, and limited mix of retail use in town centres must be addressed to limit the appearance of gambling outlets (Lopez et al., 2016), there appear to be no specific restrictions on tackling existing clusters of outlets. A proposal to limit harm from existing outlets may include restrictions on fixed odds betting terminals (FOBT) which can be a particularly damaging form of gambling. Restrictions could be introduced on stakes, speed of play and device numbers in betting outlets (Hanrahan, 2013); more recently the UK government acknowledged that they are consulting on FOBT maximum bets being reduced from £100 to between £2 and £50 ([Bibr bib86][Bibr bib86] Government, 2017).

For policy related to distributions of alcohol, fast food, tobacco and gambling outlets beyond Scotland and internationally, restrictions vary considerably (Freeman, 2014, Gainsbury et al., 2014, Hodge et al., 2008, Howard et al., 2014, Luke et al., 2016, Rehn, 2004) however oversupply of these products/services appears to be a common theme within deprived areas in the US, Canada, Australia etc. (Chaiton et al., 2013, Hay et al., 2009, Thornton et al., 2016, Wilson et al., 2006); much discussion over context specific legislation is necessary. The need for an alternative to the placement of *‘environmental bads’* within deprived areas is great. These neighbourhoods could benefit from the ‘smart growth’ approach where experts in planning, building, transportation and public health work to improve residents’ quality of life, and promote healthy behaviours, through policies which encourage for example mixed land and building use and a more diverse set of retail resources (United States Environmental Protection Agency, 2017).

### 4.4 Strengths

This study displayed a number of strengths; we used a novel application of a method to explore socio-spatial distribution of retail outlets which provided objective, robust detection of outlet clusters and accompanying statistical data. Limited research made used of a spatial scan statistic to locate clusters of alcohol outlets in Texas (Han and Gorman, 2014). Prior research has often been restricted by calculating and comparing densities within existing administrative boundaries (Chaiton et al., 2013, James et al., 2017, Livingston, 2012, Loomis et al., 2013, Wilson et al., 2006); this can be problematic due to the ‘modifiable areal unit problem’, i.e. when arbitrarily classified units such as postal/zip codes or census tracts are used to report spatial patterning resulting in potential statistical bias (Openshaw, 1984). The method used here is less restricted by pre-defined boundaries as it locates clusters of outlets across the whole city. Furthermore, while previous work explored one or two categories in isolation our study included a number of categories of health damaging outlets, allowing for the location of areas with greater access to a number of unhealthy products and services and contributes to the literature on determinants of the co-occurrence of unhealthy behaviours (Meader et al., 2016).

### 4.5 Limitations

Due to the large numbers of outlets contained within the database we did not validate every outlet. Although we cannot assume that all data within the database is accurate we have no specific reason to believe that bias has occurred due to missing/incorrect outlet data being more or less likely in areas of a particular level of deprivation. Nonetheless future work which involved a level of in-situ validation would provide added benefit. A study based in Minnesota, US created a modified ‘ground-truthing’ technique (i.e. checking accuracy using on the ground observation) by exploring patterns of error in outlet data and a focus on validation of central commercial clusters specifically; findings showed that this technique provided a high level of accuracy at a lower cost than traditional ground-truthing (Caspi and Friebur, 2016). Our study explored the availability of unhealthy resources but did not provide comparative analysis exploring access to health beneficial resources such as fruit and vegetable retailers, or supermarkets (Lamichhane et al., 2013). We cannot say whether the areas with higher densities of tobacco, alcohol, gambling and fast food outlets are compensated by better access to ‘healthy’ resources, although previous Scotland based work did not find fruit and vegetable shops, supermarkets (Macdonald et al., 2009) or sports facilities (Lamb et al., 2010) to be more accessible in deprived areas. The creation of ‘retail environment indices’ which include ratios of healthy resources to unhealthy resources (Cobb et al., 2015), may be useful in future research but beyond the capacity of the current study. This research identifies the locations of clusters of outlets but we cannot say whether higher numbers of clusters within deprived areas reflects greater population numbers (i.e. greater demand); nonetheless greater availability of ‘harmful’ products/services within more disadvantaged areas is in itself a matter of great concern. We did not apply population weighting to the cluster detection of outlets and this could be considered a limitation of the study, however detecting clusters of ‘environmental bads’ are important regardless of whether there is a lesser or greater residential population, particularly as this is complex when considering city centre non-residential areas. Indeed a higher population living in close proximity to outlet clusters may equate to a higher level of population exposure. Our study looks only at spatial access but does not explore whether those living in neighbourhoods with clusters of outlets are more likely to use local retailers as proximity does not necessarily equate to use. However previous US based work maintained that geographic presence of food outlets was correlated to individuals’ awareness of their presence (Barnes et al., 2015b) which could influence use of nearby outlets. We acknowledge that people could access gambling services on-line, could order fast food to be delivered to their homes from outlets out with their neighbourhood, and may have access to illegal sources of tobacco and alcohol (Stead et al., 2001); we do not include these sources in our study as reliable data are not readily available; nor is data on the different sizes of outlets or range of products sold. Finally, one limitation of the SaTScan™ software is that it may not identify clusters which are located on, or very close to, study area boundaries. However, by including a large boundary surrounding the Glasgow City boundary, we have avoided potential edge effects. Our previous study used UK wide data and conducted a sensitivity analysis of Glasgow boundaries, finding the results were not sensitive to boundary definition (Olsen et al., 2017).

## 5 Conclusion

We observed a greater number of clusters of *‘environmental bad’* outlets (alcohol, fast food, tobacco, and gambling outlets combined) located within more deprived areas. Additionally when analysed individually alcohol outlets, tobacco outlets, fast food outlets and gambling outlets were clustered within deprived areas. Furthermore, we found a greater number of overlapping clusters in more deprived neighbourhoods showing evidence of co-location. This research makes use of a robust technique and novel application of cluster analysis to detect clusters of outlets and adds to existing evidence that deprived areas have increased opportunities to access potentially health damaging and/or addictive goods or services. The findings reported here may aid authorities to develop policies and planning regulations appropriate for the areas in greatest need.
