# Supplementary material for: Do ‘environmental bads’ such as alcohol, fast food, tobacco, and gambling outlets cluster and co-locate in more deprived areas in Glasgow City, Scotland?
Source: Health Place. 2018 May;51:224–31. doi: 10.1016/j.healthplace.2018.04.008 (PMC5989655; doi:10.1016/j.healthplace.2018.04.008)
Supplement: Supplementary file 1 — Supplementary material [file mmc1.docx]

**Supplementary Table**

***Sup Table 1: Spatial cluster statistical information by retailer***

| **Observed** | **Expected** | **Log Likelihood ratio** | **P value** | **SIMD quintile** |
| --- | --- | --- | --- | --- |
| **a) All outlets** | | | | |
| 7 | 0.1 | 22.7 | 0.008 | 1 |
| 9 | 0.3 | 21.7 | 0.012 | 1 |
| 10 | 0.5 | 20.0 | 0.032 | 1 |
| 10 | 0.2 | 31.8 | 0.001 | 1 |
| 11 | 0.4 | 26.5 | 0.001 | 1 |
| 12 | 0.5 | 27.1 | 0.001 | 1 |
| 18 | 1.8 | 25.0 | 0.002 | 1 |
| 22 | 1.8 | 34.5 | 0.001 | 1 |
| 29 | 3.1 | 39.0 | 0.001 | 1 |
| 30 | 3.2 | 40.3 | 0.001 | 1 |
| 7 | 0.2 | 19.9 | 0.032 | 2 |
| 7 | 0.1 | 20.5 | 0.021 | 2 |
| 8 | 0.1 | 27.2 | 0.001 | 2 |
| 16 | 1.3 | 25.9 | 0.001 | 2 |
| 21 | 3.2 | 21.8 | 0.012 | 2 |
| 28 | 1.4 | 58.3 | 0.001 | 2 |
| 50 | 3.5 | 86.5 | 0.001 | 2 |
| 57 | 3.5 | 106.2 | 0.001 | 2 |
| 13 | 1.1 | 20.1 | 0.027 | 3 |
| 19 | 2.3 | 23.3 | 0.007 | 3 |
| 42 | 3.1 | 71.1 | 0.001 | 3 |
| 66 | 3.4 | 135.2 | 0.001 | 3 |
| 68 | 3.5 | 137.8 | 0.001 | 3 |
| 178 | 3.5 | 532.9 | 0.001 | 3 |
| 18 | 0.7 | 40.1 | 0.001 | 4 |
| 29 | 2.6 | 43.9 | 0.001 | 4 |
| 35 | 2.7 | 58.1 | 0.001 | 4 |
| 45 | 3.6 | 73.2 | 0.001 | 5 |
| **b) Alcohol outlets** | | | | |
| 5 | 0.0 | 18.5 | 0.046 | 1 |
| 5 | 0.0 | 20.0 | 0.023 | 1 |
| 9 | 0.4 | 19.3 | 0.032 | 1 |
| 14 | 1.4 | 19.9 | 0.024 | 1 |
| 20 | 1.8 | 29.7 | 0.001 | 1 |
| 20 | 1.8 | 30.4 | 0.001 | 1 |
| 6 | 0.1 | 19.3 | 0.033 | 2 |
| 7 | 0.1 | 20.9 | 0.011 | 2 |
| 16 | 2.0 | 19.6 | 0.028 | 2 |
| 18 | 0.7 | 41.8 | 0.001 | 2 |
| 31 | 1.8 | 58.6 | 0.001 | 2 |
| 50 | 2.3 | 108.1 | 0.001 | 2 |
| 11 | 0.7 | 20.3 | 0.021 | 3 |
| 26 | 1.7 | 46.6 | 0.001 | 3 |
| 55 | 2.1 | 127.4 | 0.001 | 3 |
| 148 | 2.2 | 484.1 | 0.001 | 3 |
| 16 | 1.3 | 25.3 | 0.003 | 4 |
| 29 | 1.4 | 60.8 | 0.001 | 4 |
| 40 | 1.9 | 83.6 | 0.001 | 4 |
| 37 | 2.3 | 68.9 | 0.001 | 5 |
| **c) Fast food outlets** | | | | |
| 8 | 0.1 | 24.6 | 0.002 | 1 |
| 11 | 0.6 | 21.8 | 0.006 | 1 |
| 10 | 0.2 | 28.1 | 0.001 | 1 |
| 14 | 1.0 | 24.7 | 0.002 | 1 |
| 14 | 0.4 | 36.7 | 0.001 | 1 |
| 16 | 1.1 | 27.6 | 0.001 | 1 |
| 4 | 0.0 | 17.3 | 0.044 | 2 |
| 14 | 0.6 | 31.8 | 0.001 | 2 |
| 17 | 0.8 | 36.8 | 0.001 | 2 |
| 28 | 1.1 | 64.2 | 0.001 | 2 |
| 9 | 0.3 | 20.8 | 0.009 | 3 |
| 17 | 0.8 | 35.6 | 0.001 | 3 |
| 20 | 1.2 | 38.5 | 0.001 | 3 |
| 61 | 1.1 | 185.9 | 0.001 | 3 |
| 15 | 0.9 | 28.3 | 0.001 | 4 |
| 14 | 1.1 | 22.9 | 0.003 | 5 |
| **d) Tobacco outlets** | | | | |
| 5 | 0.0 | 19.4 | 0.017 | 1 |
| 6 | 0.1 | 20.9 | 0.007 | 1 |
| 9 | 0.5 | 18.0 | 0.036 | 1 |
| 12 | 0.8 | 20.9 | 0.007 | 1 |
| 13 | 1.3 | 18.1 | 0.036 | 1 |
| 13 | 0.8 | 24.1 | 0.002 | 1 |
| 13 | 0.7 | 26.7 | 0.001 | 1 |
| 15 | 0.4 | 40.5 | 0.001 | 2 |
| 25 | 1.3 | 50.3 | 0.001 | 2 |
| 14 | 1.2 | 21.9 | 0.005 | 3 |
| 18 | 1.2 | 31.5 | 0.001 | 3 |
| 23 | 1.2 | 45.7 | 0.001 | 3 |
| 50 | 1.3 | 134.7 | 0.001 | 3 |
| 18 | 1.0 | 35.8 | 0.001 | 4 |
| 22 | 1.3 | 41.3 | 0.001 | 5 |
| **e) Gambling outlets** | | | | |
| 7 | 0.2 | 16.9 | 0.016 | 1 |
| 10 | 0.4 | 23.8 | 0.001 | 1 |
| 5 | 0.0 | 20.9 | 0.002 | 2 |
| 7 | 0.1 | 24.1 | 0.001 | 3 |
| 10 | 0.3 | 25.2 | 0.001 | 4 |

*Note: Table contains the location of all statistically significant (p<0.05) outlet clusters in Glasgow. The likelihood ratio test provides evidence of the elevated risk of an outlet in that location. Observed value represents the total number of retail outlets within geographical window (cluster); Expected value represents the expected number of retails outlets within each geographical window at each location following gradually scanning a window across space.*
